# Is telomere length associated with mate choice in a songbird with a high rate of extra-pair paternity?

**DOI:** 10.1371/journal.pone.0182446

**Published:** 2017-08-04

**Authors:** Arild Johnsen, Angela Pauliny, Jan T. Lifjeld, Donald Blomqvist

**Affiliations:** 1 Natural History Museum, University of Oslo, Oslo, Norway; 2 Department of Biological and Environmental Sciences, University of Gothenburg, Gothenburg, Sweden; University of Missouri Columbia, UNITED STATES

## Abstract

Telomere length is related to aging in many eukaryotes and the rate of telomere attrition has been suggested to reflect individual genetic quality. Telomere length could thus have implications for mate choice. We investigated telomere length variation in bluethroat *Luscinia svecica* families with mixed paternity, including social parents, extra-pair fathers and nestlings, testing whether telomere length is associated with social and/or extra-pair mate choice through assortative mating or selection of mates with relatively long telomeres. In adults, relative telomere length (rTL) did not differ between the sexes, nor between two age categories. In chicks, however, rTL decreased with body mass at sampling (an index of nestling age). We found a positive correlation between the rTL of social mates, suggesting assortative mating with respect to telomere length or a correlative thereof. However, extra-pair males did not differ from social mates in rTL, and accordingly there was also no difference between within- and extra-pair young (i.e. half-siblings) when controlling for the effect of mass. We found no relationships between telomere length, age and fitness-related traits in adults, but an intriguing year-difference in telomere length in both sexes. In conclusion, we found no support for the idea that females choose extra-pair males based on their telomere length, but social mate choice seems to be influenced by rTL, possibly through its co-variation with aspects reflecting individual quality, like early arrival at the breeding grounds.

## Introduction

Mate choice is a prominent feature of most living organisms, and may strongly influence the rate of evolution through the forces of sexual selection [1]. In animals, mate choice may be based on a variety of biometric (e.g. secondary sexual traits, size), chemical (e.g. smell, properties of gametes and/or seminal fluid) or environmental characteristics (e.g. features of the territory of the potential mate), which may reflect the genetic quality and/or the genetic compatibility of the potential mate. Evidence for mate choice on aspects of individual genetic constitution is building up, including choice based on major histocompatibility genes in vertebrates (reviewed in [2]), and on overall genetic similarity in birds (reviewed in [3, 4]). Mating behaviour includes copulations outside the social pair bond (extra-pair copulations), potentially leading to mixed parentage in a brood or litter (extra-pair fertilizations). In birds, for example, extra-pair paternity is frequent [5].

One aspect of individual genetic quality that may be targeted by both within-pair and extra-pair mate choice is telomere length. Telomeres consist of short, tandemly repeated DNA sequences that are part of a protective cap at the end of eukaryotic chromosomes. Their many vital functions in the cell are length-dependent, and telomere attrition is thus associated with processes of cell death and organismal senescence [6]. Overall, somatic telomeres shorten with age in many vertebrate species [7] and several studies have found that telomere length predicts longevity [8–11], although the causality of this relationship has been questioned (e.g. 12, 13]). The rate of telomere length reduction has been shown to be correlated with a variety of stress factors, including psychological stress, infection, rapid growth and reproduction [14–19], and to reflect individual phenotypic quality [8, 20]. Furthermore, recent work has documented associations between telomere length, inbreeding and behaviour [21, 22]. The relationship between telomeres and fitness, including both reproductive success and life span (e.g. [8, 11]), opens up the possibility for mate choice based on this genetic feature, the benefit of which could be either direct (e.g. higher territory quality or better parental care) or indirect (e.g. increased offspring telomere length). To date, however, the possible association between telomere characteristics and mate choice has not been explored.

Assuming that females are unable to assess telomere length directly, there may be some trait reflecting telomere length on which they could base their choice. Given the above mentioned relationships with fitness and individual quality, potential correlates of telomere length that could be used in mate choice include age and overall condition/viability, which again could be reflected in secondary sexual characters. In passerines, many studies have found that older males are more likely to sire offspring through extra-pair copulations than younger males (reviewed in [23]), a pattern that could be due to female choice for older males and/or superior competitive skills or experience with obtaining extra-pair copulations among older males. Assuming a heritable component of initial male telomere length, as found in several human studies (reviewed in [24]), we hypothesized that extra-pair offspring should have longer telomeres than their within-pair half-siblings if extra-pair fathers are generally older and of higher quality than within-pair males. The rationale for this is that in the group of old males, all might have genes for longevity, whereas among the young males, there will be some that do not and are destined to a short life. Additionally, data from humans have shown a striking difference between telomeres in somatic tissue, that generally shorten with age, and sperm telomeres that increase with age, presumably because of the high enzymatic activity of telomerase in sperm-producing tissue [25, 26]. There is also evidence that offspring of old human males have longer leucocyte telomeres than those with younger fathers [26, 27]. Accordingly, females could improve one aspect of genetic quality, i.e. telomere length, in their offspring by choosing older males.

The bluethroat *Luscinia svecica* is a small (~18g), predominantly socially monogamous, migratory passerine with a high frequency of extra-pair paternity; up to 40% extra-pair offspring in some years [28, 29]. Experimental studies have shown that both social and extra-pair mate choice is influenced by the appearance of the male throat ornament [30–32], while under natural conditions, male age seems to be a more important determinant of paternity, with old males being more successful than young ones [33]. Extra-pair mating conveys genetic benefits in the form of compatible genes to females in this species, as shown by a higher immune response of both maternal and paternal half-siblings arising from extra-pair copulations [34, 35], higher multilocus heterozygosity for extra-pair offspring and a concomitant lower genetic similarity between extra-pair mates than within-pair mates [34].

In this study, we determine relative telomere length (rTL) for adults and offspring in 40 bluethroat families characterized by having mixed paternity and at least one extra-pair sire identified. Our aim was to examine whether telomere length is associated with social and genetic mate choice. In particular, we (1) tested for assortative mating with respect to rTL among the social pair members, (2) examined whether extra-pair males differ from within-pair males in rTL and, accordingly, whether extra-pair offspring differ from their within-pair half-siblings, and (3) examined correlations between rTL and fitness-related traits in adults.

## Materials and methods

### Fieldwork

Field work was carried out at the Øvre Heimdalen field station, in Øystre Slidre, Oppland Norway (61°25N, 8°52E), during May–July in 1998 and 1999. The individuals analysed in the present study represent a subset of individuals sampled for a genetic parentage analysis that has been published previously [33–35]. We selected 40 broods (201 nestlings) with mixed paternity, together with their social parents and at least one identified extra-pair sire (see [34, 35]). Basic field procedures and details of the genetic parentage analysis can be found in those previous papers and are only briefly summarized here. Adults were caught in mist nest, using playback (most males), or at the nest (most females). The birds were aged as young (1 year old) or older according to Svensson [36]. A blood sample (brachial venipuncture) and biometric measurements (mass, wing length and tarsus length) were collected from both sexes. In addition, we measured the width of the chestnut breast band (with a calliper, 1999 only) and the colour reflectance of the upper blue chin feathers (with a spectroradiometer, 1998 only) in males. From the reflectance measurements, we calculated estimates of brightness, hue and chroma (see [33] for details). We scored the colourfulness of females on a scale from 1–10 [37]. Nestlings from nests found before hatching were blood sampled by brachial or metatarsal venipuncture on day 2 post-hatch (day 1 in one case), and those from nests found after hatching were bled immediately after finding the nest, resulting in the age of nestlings (estimated based on their mass; [38]) ranging from 1–10 days old. We measured tarsus length for nestlings surviving until day 8 post-hatch (133 nestlings in 28 nests). Catching and ringing bluethroats was approved by Norwegian bird ringing authorities. No permit was required for blood sampling at the time of sampling. Blood samples collected from adults and nestlings were stored at 4°C in Queens lysis buffer [39] until further analyses.

### Parentage and heterozygosity

Originally, DNA was extracted using the QIAamp (Qiagen) blood extraction kit. We amplified up to 11 microsatellite loci using PCR (see [34] for details), and calculated multilocus standardized heterozygosity for adults according to Coltman et al. [40]. The parentage analysis was based on 4–11 loci (average: 8.78; [34]) and offspring were considered extra-pair if they showed two or more mismatches with their social father. We used CERVUS [41] to assign fathers [34].

### Telomere analyses

Building on Cawthon [42], we used quantitative real-time PCR (qPCR) to measure relative telomere length (rTL). To ensure high quality and transparency in our qPCR assays, we followed applicable MIQE guidelines [43]. Analyses were based on our previously described protocols [16, 44], which we adapted for amplification of bluethroat DNA.

In brief, we freshly extracted genomic DNA in 2015 from aliquots of the whole blood samples collected in 1998 and 1999 for other analyses (see above), using the Omega EZNA blood kit. A subset of DNA extracts were tested on agarose gels and their DNA integrity proved to be intact. The quantity and quality of the extracted genomic DNA was assessed in duplicate on a NanoDrop spectrophotometer (A_260/280,_ mean ± SE = 2.07 ± 0.01, n = 370). Based on the average concentration, each sample was diluted to 0.25 ng μl^-1^ with autoclaved Milli-Q water. We performed relative quantification as described by Pfaffl [45], in which the amplification of telomeric repeats (T) is first normalized to that of a reference gene (S). Relative telomere length of a focal sample was then obtained by relating its T/S ratio (i.e. telomeric content per genome) to that of a calibrator sample. Two such samples were analysed in triplicate on all PCR plates, one of which was randomly picked for the above relative quantification.

Amplifications were carried out on a CFX Connect Real-Time PCR Detection System (Bio-Rad) using clear-well plates and adhesive optical seals (both Bio-Rad). We used previously published primer sequences [46] to amplify telomeric repeats (Tel1b, 5’-CGG TTT GTT TGG GTT TGG GTT TGG GTT TGG GTT TGG GTT-3’; Tel2b, 5’-GGC TTG CCT TAC CCT TAC CCT TAC CCT TAC CCT TAC CCT-3’), and glyceraldehyde-3-phosphate dehydrogenase (GAPDH) as the reference gene (GAPDH-F, 5’-AAC CAG CCA AGT ACG ATG ACA T-3’; GAPDH-R, 5’-CCA TCA GCA GCA GCC TTC A-3’). Both PCR protocols were first optimized for use in bluethroats by using the qPCR machine’s gradient function as well as testing a range of primer concentrations (data not shown). Each PCR reaction contained 1ng sample DNA in a total of 10μl 1x SsoAdvanced Universal SYBR Green Supermix (Bio-Rad). We used final concentrations of 100nM and 200nM for the forward and reverse telomere primers, respectively, or alternatively 175nM each for GAPDH primers. Reaction conditions included an initial denaturation at 96°C for 3 min, followed by 25 (telomere) or 40 (GAPDH) cycles of 96°C for 15 sec and 56°C (telomere) or 60°C (GAPDH) for 45 sec. After each run was completed, a melt curve (55/59°C—96°C, 0.5°C increase cycle^-1^) was generated to confirm PCR specificity. To avoid potential sample degradation, for example due to repeated freeze-thawing cycles, diluted samples were prepared as single-use aliquots, and analysed within one month. In addition, we prepared similar single-use aliquots for the calibrator samples, all primer solutions, and autoclaved MilliQ water. Corresponding telomere and GAPDH amplifications were carried out on different plates (but same well position) and immediately after each other on the same day, using the same sample aliquot. We analysed all focal samples in triplicate and used average values in subsequent analyses. Each bluethroat family, including the mother and her offspring, as well as the within-pair (tending) father and all extra-pair fathers, was placed on the same qPCR plate for optimal comparison. All families were, however, randomly allocated to one of the 13 plates. In addition to the calibrator and focal samples, a negative control (no template control, NTC) was included on all plates in triplicate. None of these NTCs for either the telomere or GAPDH amplifications ever resulted in a fluorescence signal above the baseline threshold (set automatically by the CFX Manager Software version 3.1, Bio-Rad). The intra-plate coefficient of variation (samples run in triplicate) ranged between 0.056%–3.64% (telomere), and 0.018%–1.37% (GAPDH). The inter-plate coefficient of variation (based on the two calibrator samples run in triplicate on all plates of the present study) ranged between 1.80%–1.87% (telomere), and 0.73%–0.75% (GAPDH).

To determine the amplification efficiency of each qPCR assay, needed in the mathematical model for relative quantification by Pfaffl [45], we followed recommendations by Svec et al. [47]. Thus, for each amplification target, two independent estimates of the PCR efficiency were obtained by analysing 6 serial 1:5 dilutions of an equal mix of 2 focal samples in quadruplicate (15 ng—0.0048 ng DNA per well). Standard curves were generated by the CFX Manager Software, and PCR efficiencies (E) calculated as E = 10 ^[-1/slope]^. The average PCR efficiency and R^2^ for the telomere and GAPDH amplification was 96.9% and 0.980, and 102.7% and 0.995, respectively.

### Statistical analyses

Some adults bred in both years, but we only extracted DNA from one blood sample per individual and hence only included the rTL estimate from the year of blood sampling in the analyses. For this reason, and because of some missing measurements, sample sizes vary in the different analyses involving adults. Adult rTL were normally distributed for both sexes (Shapiro-Wilk tests, both *p* > 0.24), and the same was true for nestling rTL after log transformation (*p* = 0.59).

For a subset of adult males (*n* = 13), we obtained two independent estimates of rTL (i.e. run in separate PCRs and on separate plates, but based on the same DNA extract). The two estimates were highly correlated (*r* = 0.82, *p* = 0.0006), testifying to the reliability of our estimates of rTL.

In analyses in which adult rTL was used as response variable, we employed parametric tests (Pearson correlation, Welsh two-sample t-tests adjusting for unequal variances, paired t-tests, ANOVA, ANCOVA). In pairwise comparisons of within-pair versus extra-pair male rTL, we included both dyads in cases where two extra-pair males had been identified (*n* = 5 nests). To test for possible differences related to the different qPCR runs, we performed ANCOVAs with rTL as response variable and year, plate number and their interaction as independent variables. However, neither plate number nor its interaction with year showed any significant effect in either sex (all *p* > 0.22). We performed three-way ANOVAs to test for year effects and differences between the sexes and age groups, including three-way and two-way interaction terms in the initial model. Year was also added in ANCOVAs testing for (1) relationships among social mother, social father and extra-pair fathers in telomere length, and (2) relationships between rTL and fitness-related traits. In the latter models we also added the interaction between age and the various traits in the initial models, since several of the trait are known to be age-related. Only interaction terms with *p* < 0.10 were retained in the final models (and are thus presented).

We performed linear mixed models with REML in the LmerTest package, to test for differences between half-siblings in rTL (response variable), using paternity as fixed factor and nest ID as random factor, and to test for relationships between offspring and parental rTL using nest ID/genetic father ID as random factor. One brood was excluded from these analyses (but included in the comparisons involving adults), since we failed to obtain an estimate of rTL from the only within-pair nestling in the brood. Nestling mass had a significant effect on telomere length (see Results), and we therefore added mass at the time of bleeding as a co-variate. This reduced the sample size further to 182 offspring in 36 nests, since we lacked data on nestling mass for 16 offspring. All analyses were done in R 3.2.3 (R Development Core Team, Vienna, Austria).

## Results

**Fig 1:**
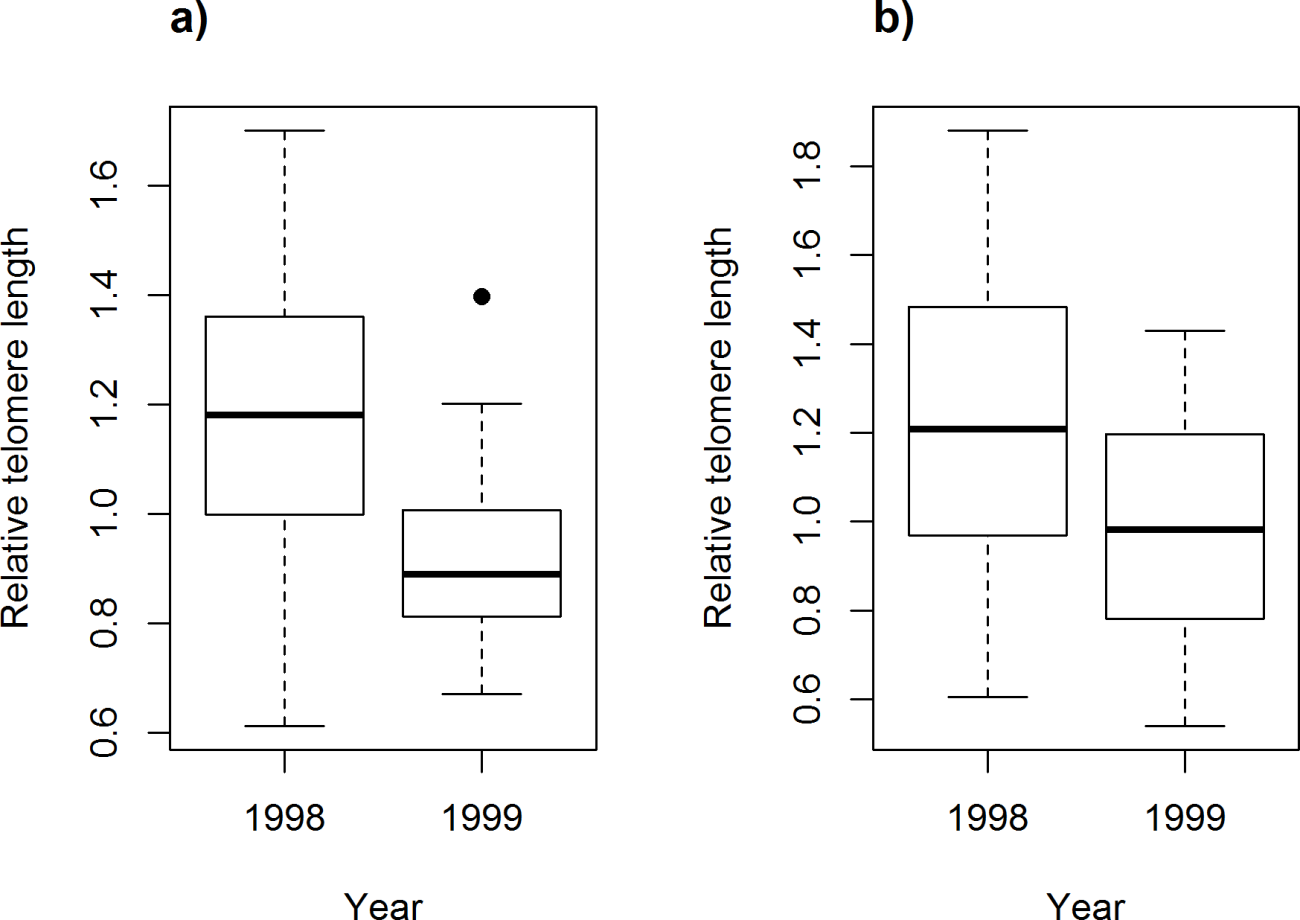
**Box and whisker plot showing year-differences in relative telomere length for (a) adult males and (b) adult females**. The horizontal line represents the median, the box is defined by the lower and upper quartiles and the whiskers extend to the highest and lowest values, except outliers (black dots).

For both sexes, telomeres were on average longer in 1998 than in 1999 (Fig 1). Including the year effect, the two sexes did not differ significantly in rTL and there were no significant differences between young and older birds in either sex (three-way ANOVA, response variable: rTL, independent variables: sex: *F*_1, 93_ = 0.63, *p* = 0.43, estimate ± SE (female vs. male) = 0.06 ± 0.06; age: *F*_1, 93_ = 0.31, *p* = 0.58, estimate ± SE (old vs. young) = 0.04 ± 0.06; year: *F*_1,93_ = 16.4, *p* < 0.001, estimate ± SE (1999 vs. 1998) = -0.24 ± 0.06; *n* = 12 young and 43 older males, 15 young and 27 older females).

**Fig 2:**
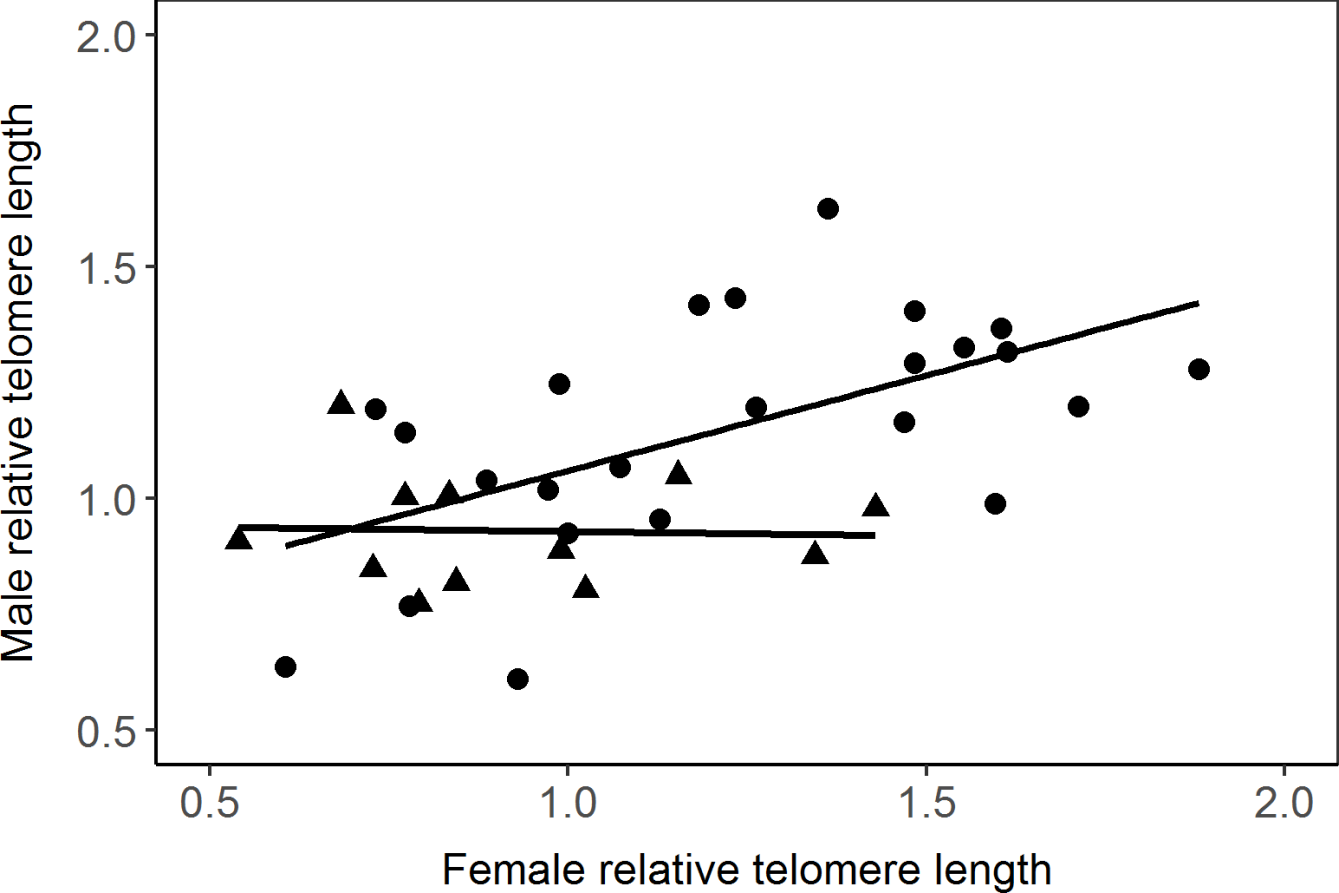
Relationship between the relative telomere length of females and their social mates (= 36 pairs), shown separately for the two years of study (1998: Black circles, 1999: Black triangles). The lines represent the linear regression lines.

Within social pairs, we found a positive relationship between the rTL of males and females (ANCOVA, response variable: rTL of male, independent variables: rTL of female: *F*_1, 32_ = 18.3, *p* = 0.0002; year: *F*_1, 32_ = 3.1, *p* = 0.09; interaction: *F*_1, 32_ = 3.3, *p* = 0.08; *n* = 36 pairs; Fig 2). Looking at each year, there was a relatively strong correlation between pair mates in 1998 (*r* = 0.58, *n* = 24 pairs), but not in 1999 (*r* = -0.04, *n* = 12 pairs). In order to test whether the correlation in rTL between social mates could be an artefact arising from analysing pairs on the same sample plates, we repeated the analysis on eight pairs where we had a second estimate for the same male from another plate. Again, there was a positive correlation of a similar magnitude, albeit not significant due to the small sample size (*r* = 0.47, *p* = 0.23, *n* = 8 pairs). Similarly, we found an almost significant positive relationship between the rTL of females and their extra-pair mates (ANCOVA, response variable: rTL of extra-pair male, independent variables: rTL of female: *F*_1, 37_ = 4.0, *p* = 0.052; year: *F*_1, 37_ = 9.0, *p* = 0.005; *n* = 40 female-extra-pair male dyads).

**Fig 3:**
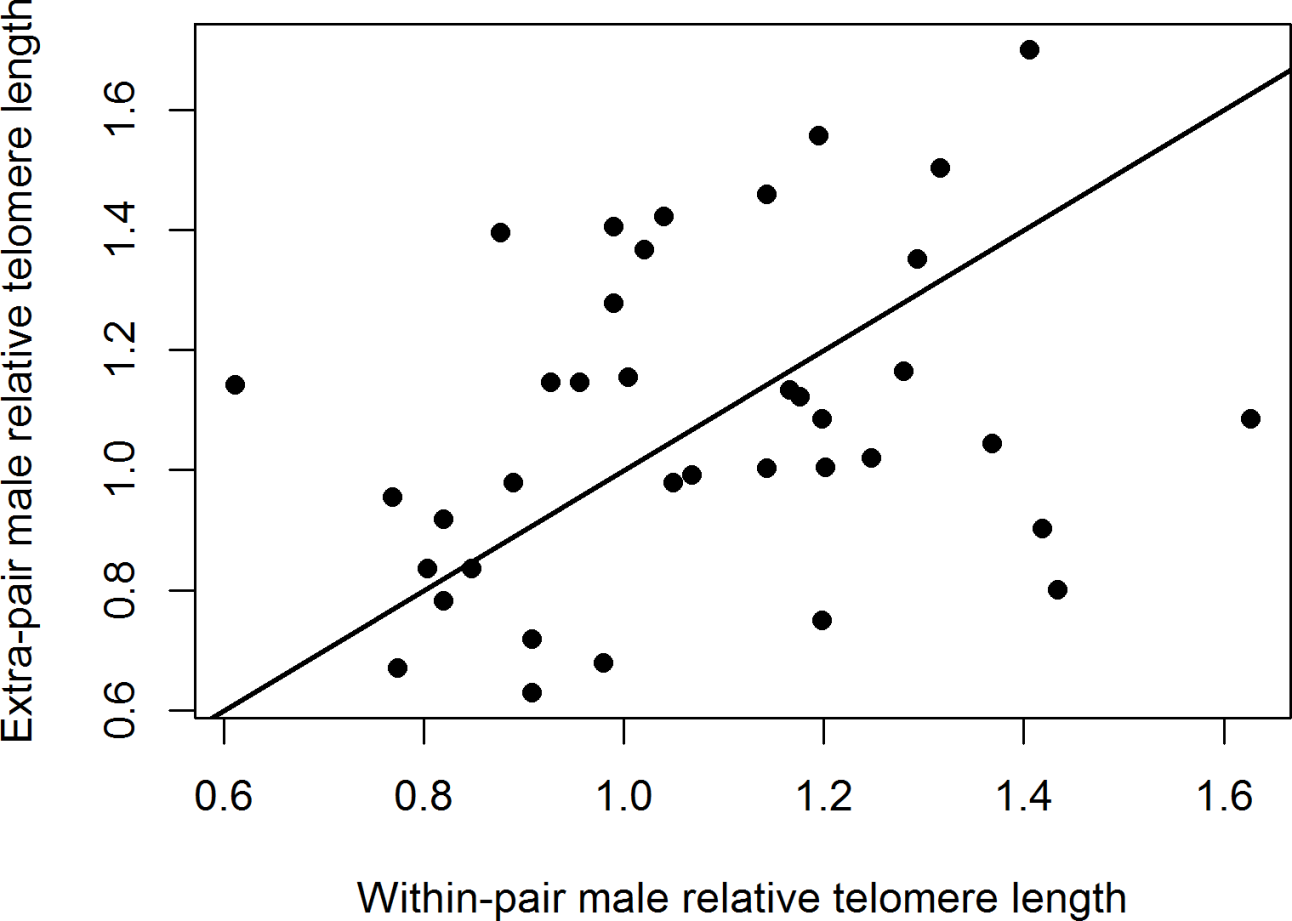
Relative telomere length of within-pair males versus the males that cuckolded them (*n* = 38 pairwise comparisons). The line represents the line of unity.

**Fig 4:**
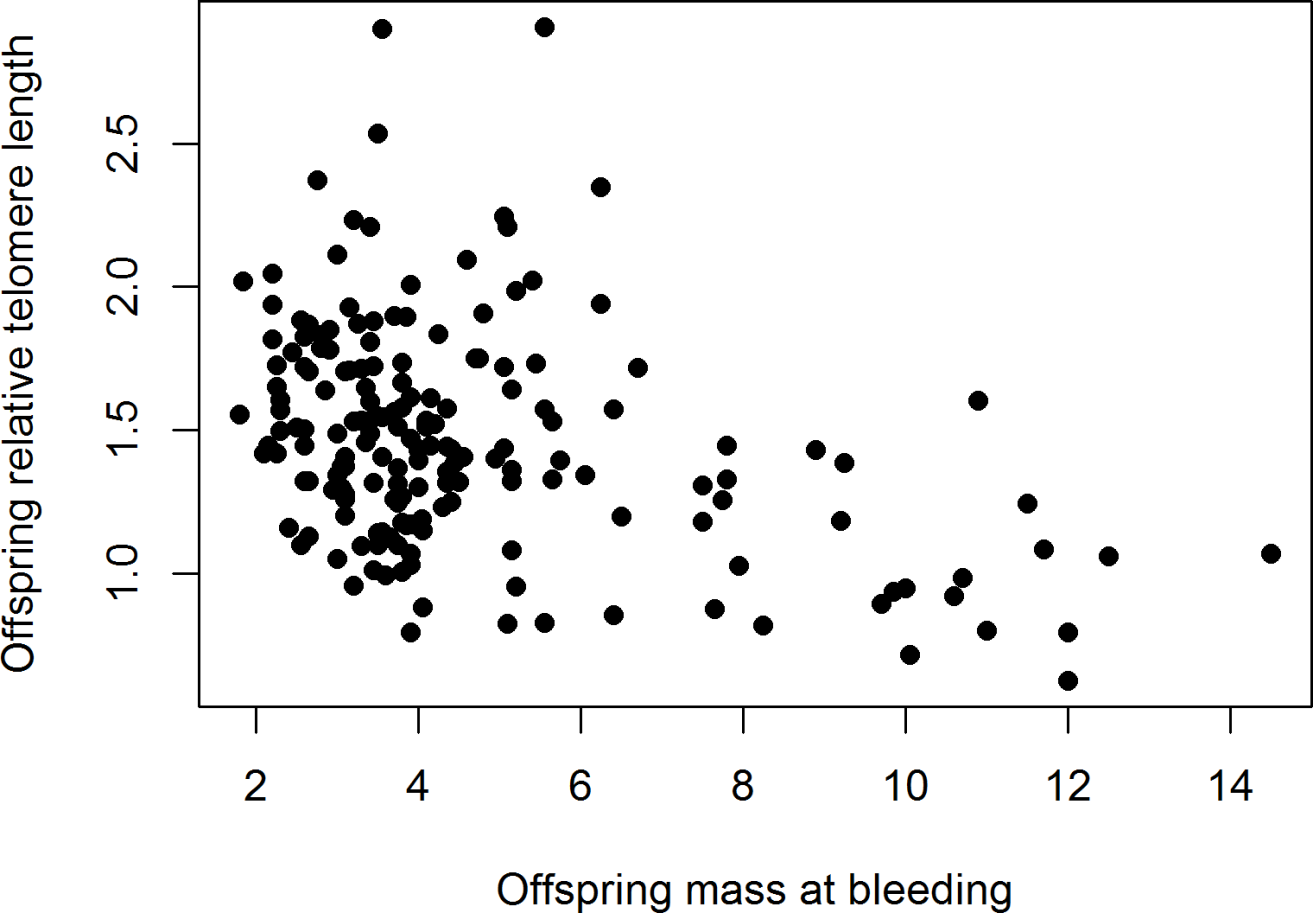
Offspring relative telomere length plotted against their mass at the time of bleeding.

There was no significant difference between the rTL of extra-pair males and the males they cuckolded (paired t-test, *t*_37_ = 0.16, *p* = 0.88; Fig 3). Consistently, paternity (within-pair or extra-pair) was not significantly related to rTL in the offspring (linear mixed model, paternity: *t*_161.1_ = -0.86, *p* = 0.39, estimate ± SE = -0.04 ± 0.05; *n* = 182 offspring in 36 nests with mixed paternity). There was no year-effect on the rTL of offspring (*t*_32_ = -0.60, *p* = 0.55, estimate ± SE = -0.05 ± 0.09). However, the model did reveal a significantly negative relationship between nestling mass (a proxy for nestling age) and their rTL (*t*_58.4_ = -3.5, *p* = 0.0008, estimate ± SE = -0.06 ± 0.02; Fig 4). In the subset of nestlings that survived until day 8, tarsus length was negatively related to rTL (*t*_100.7_ = -2.99, *p* = 0.004, estimate ± SE = -0.06 ± 0.02). Controlling for nestling mass, nestling rTL tended to be positively related to the rTL of their mother (t_32.8_ = 1.81, p = 0.08, estimate ± SE = 0.25 ± 0.14), but not to that of their genetic (*t*_49.2_ = 0.76, *p* = 0.45, estimate ± SE = 0.11 ± 0.14) or social father (*t*_28.5_ = 0.40, *p* = 0.69, estimate ± SE = 0. 071 ± 0.18).

We found no significant associations between rTL and male or female fitness-related traits (Table 1). Testing for year-differences in traits related to reproductive effort, there was no significant difference in clutch size (*t*_33.0_ = -0.43, *p* = 0.67), but females started laying on average 3.5 days earlier in 1998 than in 1999 (*t*_38.1_ = -2.15, *p* = 0.038).

**Table 1:** Tests of associations between adult relative telomere length and fitness-related traits.

Sex	Trait	Test statistic	*p*	Estimate ± SE	*n*
Male	Wing length^a^	*F*_1, 49_ = 4.16	0.047	-0.09 ± 0.05	54
	Tarsus length	*F*_1, 51_ = 0.26	0.65	-0.007 ± 0.04	54
	Body mass	*F*_1, 51_ = 1.01	0.32	0.007 ± 0.04	54
	Chestnut band width	*F*_1, 17_ = 0.93	0.35	-0.02 ± 0.02	19
	Brightness	*F*_1, 29_ = 0.44	0.51	-0.003 ± 0.004	31
	Hue	*F*_1, 29_ = 0.03	0.87	0.001 ± 0.007	31
	Chroma	*F*_1, 29_ = 1.75	0.20	-0.47 ± 0.35	31
	Standardized heterozygosity	*F*_1, 52_ = 0.03	0.87	0.14 ± 0.24	55
	Total fertilization success^b^	*F*_1, 46_ = 0.001	0.97	-0.003 ± 0.009	49
Female	Wing length	*F*_1, 38_ = 2.14	0.15	0.04 ± 0.03	41
	Tarsus length^c^	*F*_1, 37_ = 0.98	0.33	0.18 ± 0.07	41
	Body mass	*F*_1, 38_ = 0.86	0.36	0.03 ± 0.05	41
	Colour score	*F*_1, 39_ = 0.42	0.52	0.02 ± 0.03	42
	Standardized heterozygosity	*F*_1, 39_ = 0.43	0.51	0.28 ± 0.30	42
	First egg date^d^	*F*_1, 36_ = 1.48	0.23	-0.07 ± 0.04	39
	Clutch size	*F*_1, 35_ = 0.26	0.61	-0.03 ± 0.08	38

All tests control for the effect of year (all p < 0.07) and include interaction terms for year (except male colour variables, measured in one year only) and age for each trait in the initial models. None of the p-values were significant after controlling for false discovery rate [48].

^a^ Interaction term wing length x male age retained in model, *p* = 0.09

^b^ Within-pair plus extra-pair paternity

^c^ Interaction term tarsus length x year retained in model, *p* = 0.08

^d^ Standardized between the years

## Discussion

Our results show that social pair bonds in the bluethroat are formed between individuals with similar telomere lengths. Interestingly, females did not preferentially engage in extra-pair copulations with males that had longer telomeres than their social mates and, consistently, we could not detect a difference in rTL between half-siblings. There was no indication that rTL reflected age or fitness among adults, but there was a strong negative relationship between chick mass (reflecting age) and their rTL. Adult birds of both sexes breeding in 1998 had longer telomeres than those breeding in 1999.

The correlated rTLs of females and males in social pairs (and tendency for the same with respect to the extra-pair males) are suggestive of assortative mating with respect to rTL or, perhaps more likely, a co-varying trait. For example, there is evidence for age-assortative mating in the bluethroat, including the present data set (Johnsen and Lifjeld, unpublished data). However, we could not detect a difference in rTL between younger and older adult birds, possibly because our age-classes were too coarse, or age-independent inter-individual variation in rTL was too large to reveal an effect of age on rTL. Alternatively, similar rTLs in social pair members could arise from assortative mating based on some other quality or condition-dependent trait which co-varies with rTL, such as arrival time at the breeding grounds. In other words, the pattern might not be related to any active choice of mates, but could be an effect of pairs forming among birds arriving at approximately the same time at the breeding grounds. We do not have data on arrival date for these birds, but laying date, a possible proxy for arrival date, was unrelated to female rTL. However, both sexes had longer telomeres and started to breed earlier in 1998 than in 1999, which is consistent with the possibility that assortative mating based on arrival date may result in similar rTLs of social mates. Further studies are needed to clarify the mechanisms behind the pattern we have documented here. A study of common terns (*Sterna hirundo*) found that individuals arriving early had shorter telomeres than those arriving later [20]. Irrespective of the direction, as long as the relationship between rTL and arrival date is the same between the sexes, and pairing occurs shortly after arrival to the breeding grounds, a pattern of apparent assortative mating with respect to rTL will arise.

We found no difference between the rTL of extra-pair males and the males they cuckolded, and also no difference between half-siblings. Thus, females do not seem to increase the quality of their extra-pair offspring by preferentially copulating with males with relatively long telomeres. Instead, our previous work have shown that females copulate extra-pair with genetically dissimilar males, resulting in more heterozygous and immune-responsive offspring [34, 35]. The lack of extra-pair mate choice on telomere length is consistent with our finding that offspring telomere length tended to be correlated with that of their mother, but not with that of their genetic or social father. Our study thus supports an emerging pattern of heterogametic inheritance of telomere length in birds [49–51], which may arise through contributions from additive genetic and maternal effects, as shown in the great reed warbler (*Acrocephalus arundinaceus*) [49]. In the present study, we cannot exclude the possibility that part of the telomere signal measured with qPCR stems from interstitially located repeats of the same telomeric (TTAGGG)_n_ sequence, which may be abundant in some species [52]. Although such interstitial repeats are unaffected by factors that shorten telomeres, they may potentially mask patterns with telomere length (see [24]). Even if interstitial sequences were present in the bluethroat, however, we find it unlikely that they have hampered our ability to detect parentage effects on rTL for the following reasons. First, only paternally derived interstitials would contribute, since half-siblings share the same mother. Second, to mask longer telomeres in cuckolders compared to the cuckolded males, females would have to choose within-pair males with more interstitial repeats compared to extra-pair fathers. Finally, if indeed a large proportion of the measured telomere signal was derived from interstitial repeats, the similarity in rTL between offspring and their mothers or fathers, respectively, should have been comparable (but see above).

In contrast to previous studies of relatively short-lived passerines [8, 10, 53, 54], we found no difference in rTL between young and older adult bluethroats, indicating that telomere length is relatively stable in adults of this species (maximum recorded age 11.4 years, AnAge database; [55]). It is possible that a finer resolution of the category of older birds could have revealed a decline of telomere length with age also in bluethroats, as found in many other species. Moreover, the cross-sectional data available in the present study may not accurately reflect within-individual changes in rTL. Thus, to clarify the relationship between chronological age and rTL in the bluethroat, longitudinal studies are needed.

Overall, nestlings had longer rTLs than adults, and there appeared to be a strong reduction in rTL during the first 1–2 weeks after hatching whereas there was no difference between young and older adults. The early-life reduction can be explained by the rapid growth and high number of cell-divisions that occur during the nestling period in these altricial birds. The negative relationship between skeletal size (tarsus length) and rTL in eight day old nestlings supports the idea that rapid growth comes at a cost in terms of telomere attrition [56, 57]. It should be noted that our study compares telomere length estimates across individuals, and we are therefore unable to make inferences about within-individual changes. However, the general pattern of high early-life telomere length reduction finds support in both cross-sectional and longitudinal studies of birds [8, 58–61]. The combination of a strong age-dependence of telomere length at the nestling stage and the variation in our timing of blood sampling, may have masked subtle hereditary effects on offspring telomere length related to paternity, even if we controlled for offspring mass in the analyses. Future studies of early life telomere dynamics and how they relate to paternal and maternal genetic contributions should measure telomeres at a later and standardised offspring age, when the large early-life reduction has reached its asymptote.

Telomere length is often correlated with fitness components, thereby providing an index of an individual’s quality or its biological age [8, 11, 13]. We found no significant relationships between rTL of adults and fitness-related traits, perhaps because we did not measure the most relevant of such traits. For example, Pauliny et al. [8] found correlations with lifespan in short-lived sand martins (*Riparia riparia*) and life-time reproductive success in longer-lived dunlins (*Calidris alpina*), while both of these fitness components were correlated with telomere length in sand lizard (*Lacerta agilis*) females [11]. We are, however, not able to estimate these fitness measures in bluethroats due to the low return rate of individuals to our study population (< 15% for adults, close to 0% for fledglings; Johnsen and Lifjeld, unpublished data). The significant year difference in rTL in both sexes may nevertheless indicate a quality-difference between individuals returning to the breeding site in the two years of study. The fact that females started egg-laying earlier in 1998 is consistent with the cohort from 1998 being of higher average quality than the one from 1999, especially since the weather conditions during the fertile period did not differ substantially between these two years [28]. It should be noted that most adults were captured early in the season and the year-difference in rTL should thus not be due to different conditions at the breeding site in the same year, but more likely an effect of differences in the preceding winter or the previous breeding season. Interestingly, a recent study on great reed warblers found that malarial infection, transmitted in their wintering areas, lowered the birds’ long-term fitness, apparently mediated by a faster rate of telomere attrition in infected individuals [15].

## Conclusion

Our study is the first to investigate the potential role of telomeres in mate choice. We show that female bluethroats form social pair bonds with males that have similar rTLs, which may be due to an active choice or the result of assortative mating based on a trait that co-varies with rTL. In contrast, we find that rTL does not seem to explain genetic mate choice in this species, with no difference in rTL between cuckolded males and their cuckolders, and, consistently, no difference between extra-pair and within-pair half-siblings. Our study demonstrates that associations between telomere length and mate choice may be rather complex, and future studies on a broad range of species are needed to assess the generality of our findings.
